# Safety of performing urologic elective surgeries during the covid-19 pandemic in a referential hospital

**DOI:** 10.1590/S1677-5538.IBJU.2020.0860

**Published:** 2021-01-20

**Authors:** Rui T. Figueiredo, Marina R. A. Costa, Fabricio B. Carrerette, Celso M. C. Lara, Ronaldo Damião

**Affiliations:** 1 Hospital Universitário Pedro Ernesto - HUPE/UERJ Serviço de Urologia Rio de JaneiroRJ Brasil Serviço de Urologia do Hospital Universitário Pedro Ernesto - HUPE/UERJ, Rio de Janeiro, RJ, Brasil

## INTRODUCTION

The coronavirus disease (COVID-19) is a newly diagnosed infection caused by the beta-coronavirus severe acute respiratory syndrome coronavirus 2 (SARS-CoV-2) and has become a global public health problem. The World Health Organization declared COVID-19 as a pandemic on March 11, 2020, with 507.188 deaths worldwide as of June 30, 2020. In addition to the negative impact of COVID-19 on public health, some countries faced economic difficulties due to treatment expenses and the implementation of long quarantine periods for virus containment (1).

This disease is globally challenging the public and private health sectors, which both had hospitals adapted to COVID-19 care. Most hospitals were divided into COVID-19 and non-COVID-19 areas, with different protocols, fluxes, and health teams. Moreover, temporary hospitals were built specifically for COVID-19 cases in some countries. The lack of equipment and medicine for COVID-19 treatment was a common issue worldwide, making COVID-19 more difficult to control (2).

Moreover, due to the risk of contagion, several patients with oncological disorders and other moderate and severe illnesses had their treatment interrupted or postponed. Nevertheless, research addressing the risk of COVID-19 contagion and the safety of performing elective surgical procedures for cancer and other serious diseases during a pandemic are scarce and conflicting (3, 4).

Different guidelines and protocols are proposed to perform planned surgical procedures during the COVID-19 pandemic. However, most of them include massive laboratory examinations, such as reverse transcription polymerase chain reaction (RT-PCR) or immunological tests, for COVID-19 diagnosis. When these tests are not available or scarce, a brief screening method based mainly on clinical and epidemiological history may be useful (5).

Shinder et al. described the low incidence of COVID-19 in patients submitted to surgical urologic procedures in a COVID-free hospital (6). Considering the possibility of disease progression and loss of treatment window, our hospital continued to perform urologic surgeries in patients with oncological disorders and other severe diseases throughout the -pandemic. Consequently, we used a brief screening method before patient admission and specific fluxes and protocols in the perioperative period to minimize the risk of COVID-19 contagion in planned elective surgeries.

The aim of this study was to determine the safety of performing elective planned urologic surgeries during the COVID-19 pandemic and evaluate the efficacy of our screening method, fluxes, and protocols in avoiding COVID-19 infection during hospitalization.

## MATERIALS AND METHODS

We performed a retrospective review of the medical records of all 312 patients admitted for urologic surgery in our hospital during the COVID-19 pandemic from March 11, 2020 to June 30, 2020. The study was approved by the institutional reviewer board, with register 4016785.

The present study was conducted in a public university hospital with 438 hospital beds and several specialties. The hospital became a reference for COVID-19 treatment in March 2020 and was strategic in the city public health policies and assisting patients. It is located in the second largest city, which has the second highest incidence rate of COVID-19 in Brazil. At the time of this writing, the city recorded 74.674 cases and 8.622 deaths among its 6.718.793 residents. Despite the adaptations for COVID-19, the hospital maintained some critical medical care unrelated to COVID-19, such as urologic, oncological, cardiovascular, and other emergent procedures.

The hospital was divided into areas specific for COVID-19 patients and those specific for COVID-19-negative patients with different protocols and health teams for each area. Additionally, several internal fluxes were applied to avoid contact between COVID-19-positive and COVID-19-negative patients during complementary examinations and surgical procedures. Anaesthetic protocols were elaborated, and local or spinal block anaesthesia were preferred over tracheal intubation to avoid aerosol development. Complementary exams, such as chest computed tomography scans and RT-PCR tests were performed in patients with respiratory symptoms. Moreover, a RT-PCR test was performed in symptomatic patients or routinely in patients who required intensive care unit (ICU) support postoperatively.

Patients who became COVID-19 positive during hospitalization were transferred to COVID-19 specific areas. Visitations were forbidden in COVID-19 areas and were restricted to three times a week in areas unrelated to COVID-19.

The urology service section is located in the non-COVID-19 area and is composed of four ambulatory offices, 01 infirmary with 20 beds, and 01 surgical room. During the pandemic, the aforementioned section maintained consultations and planned elective surgeries for patients with oncological disorders and other severe diseases. Furthermore, some urgent surgeries were performed. The surgical procedures were performed either in the urology operating room or in the main surgical center of the hospital, both with specific protocols and fluxes for COVID-19 and non-COVID-19 patients.

**Figure 1:**
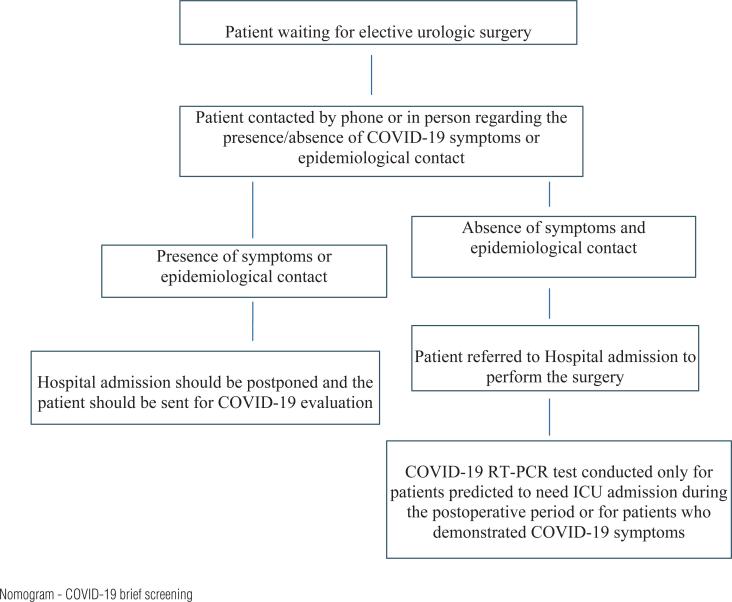
Nomogram.

Before hospital admission for planned elective surgeries, a brief screening was conducted to verify if the patient had any suspicious for COVID-19 infection. They were asked by phone or in person, regarding symptoms, including fever, cough, dyspnoea, runny nose, loss of smell, taste, or other flu-like symptoms. They were also asked for any previous contact with possible or confirmed COVID-19 positive individuals in the last 14 days. If patients were suspected for COVID-19 during this contact, they were sent to specific evaluation and had their hospital admission and surgery deferred. After admission, patients who were predicted to necessitate ICU admission postoperatively underwent a routine RT-PCR test, which had to show negative result before surgery could be performed. For emergent hospitalization, the patient's allocation to COVID-19 or non-COVID-19 areas was based on their clinical history and chest computed tomography scan results. COVID-19 Genexpert test was not routinely available during the study period (Figure-1 - screening nomogram).

During routine ambulatory return visits, patients were evaluated for the development of COVID-19 and were considered as hospital contagion cases if the disease occurred in the first 14 days after discharge. COVID-19 infection was confirmed based on clinical and epidemiological findings and RT-PCR testing during hospitalization or after discharge.

### Statistical Analysis

Categorical variables were expressed as numbers and percentages, and continuous variables as means and ranges. Due to the small number of COVID-19 carriers, statistical comparison between groups was not possible. SPSS PASW (version 22, IBM, Armonk, NY, USA) software was used for all analyses.

## RESULTS

During the pandemic, 329 surgical urologic procedures were performed on 312 patients hospitalized from March 11, 2010 to June 30, 2020. Of these, 300 (96%) underwent elective surgeries, and 12 (4%) underwent urgent procedures.

The patient's median age was 64 years, and most patients were male (83%). Arterial hypertension was present in 44% of patients, diabetes mellitus in 23%, renal insufficiency in 5%, and acquired immunodeficiency syndrome in 2% of patients. Twenty-five percent of patients had no comorbidities other than their initial disease.

The surgeries were classified as large (32%), medium (52%), and small (16%), according to surgical characteristics, most surgeries were oncological (60%). Spinal blockage was the most commonly used anaesthetic procedure (74%), followed by loco/regional anaesthesia (16%), and general anaesthesia (10%) (Table-1).

**Table 1:** Basal patient's characteristics, comorbidities, distribution of surgical procedures performed, anesthetic procedures, indication for Covid-19 RT-PCR and RT-PCR results.

Diseases and surgeries		No.	%	Patient's characteristics	No.	%
**Prostate cancer**				Mean age	64	
	Radical Prostatectomy	62	18.84	Male	273	83
	Prostate TURP	08	2.43	Female	56	17
	Orchiectomy	43	13.06	**Comorbidity**		
	HIFU	05	1.51	Arterial hypertension	145	44
	Lymphadenectomy	03	0.91	Diabetes mellitus	76	23
**Bladder cancer**				Renal insufficiency	16	5
	Radical cystectomy	03	0.91	AIDS	6	2
	Bladder TURP	42	12.76	**Surgeries**		
**Kidney cancer**				Elective	316	96
	Radical Nephrectomy	04	1.21	Emergency	13	4
**Anesthetic procedure**			
	Partial Nephrectomy	06	1.82	Spinal blockage	243	74
	Local/regional	33	10
	General anesthesia	53	16
**Urinary lithiasis**				**Indication for RT-PCR**		
	Ureterolithotripsy	31	9.42	Routine before ICU	17	89.5
	Percutaneous lithotripsy	04	1.21	Symptomatic	2	10.5
	Anatrofic lithotripsy	03	0.91	**Total**	19	100
	Cystolithotomy	03	0.91
	Double J implant	07	2.12
**BPH**				**Positive RT-PCR**		
	Retropubic prostatectomy	18	5.47	Routine before ICU	1	5.9
	Prostate TURP	22	6.68	Symptomatic	2	100
**Others**		65	19.75	**Total Positive RT-PCR**	3	15.8
**Total**		**329**	**100**	**Total in all Patients**	**3**	**0.9**

**TURP** = transurethral resection of the prostate;

**HIFU** = high-intensity focused ultrasound;

**AIDS** = acquired immunodeficiency syndrome;

**COVID-19** = coronavirus disease;

**ICU** = intensive care unit;

**RT-PCR** = reverse transcription polymerase chain reaction.

Of the 312 patients analysed, 19 (6%) were examined using nasopharyngeal RT-PCR tests for COVID-19. Routine RT-PCR tests were done in 17 asymptomatic elective patients before surgery as they were predicted to require ICU admission postoperatively. Of these patients, only one tested positive for COVID-19 and had his surgery postponed. This patient was waiting for radical nephrectomy due to a kidney tumor with caval and atrium thrombus. None of the patients who underwent elective surgeries developed COVID-19 symptoms during or after hospitalization.

Among patients who underwent urgent procedures, COVID-19 RT-PCR test was done in two patients because of respiratory symptoms, and both patients tested positive for the disease. The first patient was admitted for acute urinary lithiasis treatment and developed respiratory symptoms on the second day after admission. The second patient was admitted for severe macroscopic hematuria treatment and underwent endoscopic bladder cancer resection. She developed COVID-19 symptoms in <14 days after hospital discharge.

All patients who tested positive for COVID-19 were transferred or admitted to COVID-19 wards in the hospital and recovered after treatment.

## DISCUSSION

Maintaining elective surgery programs in a pandemic is a challenger in areas with high incidences of COVID-19 infection. Considering that currently Brazil has the third highest number of COVID-19 cumulative registered cases worldwide (5.566.049) and 766 deaths by 1.000.000 inhabitants as of November 3^rd^, a profound analysis of risks versus benefits is essential for recommending surgical procedures, mainly for oncological disorders and other severe diseases.

To minimize the risk of COVID-19 contagion, some guidelines have been proposed to face elective and urgent surgeries during a pandemic, providing protocols for COVID-19 screening and hospital fluxes, to preserve both patient and staff health. Despite some articles published on this topic, the safety of performing surgeries in such period remains unclear (7–9).

Hintze et al. assessed the impact of COVID-19 infection in patients with head and neck cancers. In this cohort, three patients were infected, and two died from COVID-19. They proposed the complete separation of COVID-19-positive and COVID-19 negative patients and dedicated COVID-19 negative staff for perioperative management (10).

Moliere et al. described the incidence of COVID-19 in 46 patients with acute postoperative respiratory symptoms, of which eight patients (17%) were diagnosed with COVID-19. Among the eight patients, five (62%) required mechanical ventilation and two (25%) died (11).

Kayani et al. established the morbidity and mortality risks for developing perioperative COVID-19 infection in orthopedic patients. This multicenter cohort study included 340 COVID-19 negative and 82 COVID-19 positive patients undergoing surgical treatment for hip fractures in Greater London, UK. In this previous trial, COVID-19 positive patients had increased postoperative mortality rates (30.5% vs. 10.3%, p <0.001) compared to COVID-19 negative patients (12).

Nahshon et al. reviewed studies involving patients who were preoperatively asymptomatic and not tested for COVID-19. Four reports were identified, comprising 64 COVID-19 asymptomatic carriers, of these, 51 carriers were diagnosed only in the postoperative period and 14 (27.5%) of patients died postoperatively (13).

Granata et al. did not report any COVID-19 infections in 12 patients who underwent urologic surgical procedures in a referral hospital during the pandemic (14). These results are conflicting, but suggest that the maintenance of some surgical procedures can be safe, with specific fluxes and protocols to reduce the risk of COVID-19 infection.

In our study, only three patients tested positive for COVID-19 in the perioperative period. Two of them were admitted less than 7 days before the positive result in the RT-PCR tests and probably were already COVID-19 asymptomatic carriers when hospitalized. The third patient tested positive less than 14 days after discharge and was considered as hospital COVID-19 infection. None of the patients who underwent elective surgeries became COVID-19 positive, and there were no deaths related to COVID-19 infection in the study patients.

The low rate of COVID-19 infection in our patients is probably due to the use of a brief screening method, hospital internal fluxes and protocols with different areas for COVID and non-COVID patients, and the anesthetic protocols, which avoided aerosol generation during the procedures. These measures may be helpful in reducing COVID-19 transmission to other patients and members of the health teams. While most guidelines recommend massive amounts of routine RT-PCR tests before elective surgeries, our study suggests that brief screening based mainly on clinical and epidemiological assessments, despite few RT-PCR tests, is effective in preventing the elective hospitalization of COVID-19 carriers and may be safe in areas with access to RT-PCR tests is unavailable or scarce.

This study has a limitation due to the small number of COVID-19 carriers diagnosed, the statistical comparison between the surgical groups was not possible.

## CONCLUSION

The risk of significant COVID-19 infection in patients who underwent elective urologic surgeries during the pandemic was low. This study implies that the use of a brief screening method with clinical epidemiological assessment is safe to avoid performance of surgeries in COVID-19 carriers. The specific fluxes and protocols probably contributed to minimizing the risk of COVID-19 contagion during hospitalization. These data may support the maintenance of essential and oncological surgical programs, even in areas with limited access to COVID-19 RT-PCR tests.
